# Biosynthesis of Silver Nanoparticles from *Protium serratum* and Investigation of their Potential Impacts on Food Safety and Control

**DOI:** 10.3389/fmicb.2017.00626

**Published:** 2017-04-18

**Authors:** Yugal K. Mohanta, Sujogya K. Panda, Akshaya K. Bastia, Tapan K. Mohanta

**Affiliations:** ^1^Biochemistry Laboratory, Department of Botany, North Orissa UniversityBaripada, India; ^2^Department of Zoology, North Orissa UniversityBaripada, India; ^3^Free Major of Natural Sciences, College of Basic Studies, Yeungnam UniversityGyeongsan, South Korea

**Keywords:** silver nanoparticles, *Protium serratum*, antimicrobial activity, antioxidant capacity, normal fibroblast cell line (L-929), food borne pathogens

## Abstract

Silver nanoparticles play an integral part in the evolution of new antimicrobials against the broad ranges of pathogenic microorganisms. Recently, biological synthesis of metal nanoparticles using plant extracts has been successfully consummated. In the present study, the biosynthesis of silver nanoparticles (AgNPs) was conducted using the leaf extract of plant *Protium serratum*, having novel ethnomedicinal. The synthesized AgNPs were characterized using UV-Visible spectroscopy, dynamic light scattering spectroscopy (DLS), Fourier transform infrared spectroscopy (FTIR), and scanning electron microscopy. The DLS study revealed the surface charge of the resulted nanoparticles that was highly negative, i.e., −25.0 ± 7.84 mV and the size was 74.56 ± 0.46 nm. The phytochemical and FTIR analysis confirmed the role of water-soluble phyto-compounds for the reduction of silver ions to silver nanoparticles. The potential antibacterial activity of AgNPs was studied against the food borne pathogens viz. *Pseudomonas aeruginosa* (IC_50_ = 74.26 ± 0.14 μg/ml), *Escherichia coli* (IC_50_ = 84.28 ± 0.36 μg/ml), *Bacillus subtilis* (IC_50_ = 94.43 ± 0.4236 μg/ml). The *in vitro* antioxidant potential of AgNPs was evaluated using 1, 1-diphenyl-2-picryl-hydrazil (IC_50_ = 6.78 ± 0.15 μg/ml) and hydroxyl radical assay (IC_50_ = 89.58 ± 1.15 μg/ml). In addition, the cytotoxicity of AgNPs was performed against fibroblast cell line L-929 to evaluate their biocompatibility. The overall results of the present investigation displayed the potential use of *P. serratum* leaf extract as a good bio-resource for the biosynthesis of AgNPs and their implementation in diverse applications, specifically as antibacterial agent in food packaging and preservation to combat against various food borne pathogenic bacteria along with its pharmaceutical and biomedical applications.

## Introduction

The phyto-mediated synthesis is a rapid, suitable and most acceptable biosynthetic method for synthesis of metal nanoparticles. Now a days, various plant parts like bark, leaf, fruit, stem and seed extracts have been successfully used for the synthesis of metal nanoparticles (Mittal et al., 2013). Among different metal nanoparticles, silver (Ag) nanoparticles have been used enormously due to their potential anti-bacterial (Mohanta et al., 2016a,b), anti-fungal and anti-proliferative activity (Kim et al., 2007; Nayak et al., 2015). Due to the excellent antimicrobial properties, the silver (Ag) nanoparticles have been extensively used in food packaging, food and seed preservation, biofertilizers, cosmetics and medicines (Marambio-Jones and Hoek, 2010; Dipankar and Murugan, 2012). Besides these applications, the silver nanoparticles were found to be implemented widely in the field of high sensitive bio-molecular detection, diagnostics, catalysis and micro-electronics (Mohanta and Behera, 2014).

A number of standard approaches by means of physical and chemical methods have been used for the synthesis of silver nanoparticles (AgNPs) viz. reduction in solutions, chemical and photochemical reactions in reverse micelles, thermal decomposition of silver compounds, radiation assisted, electro chemical, sono-chemical, microwave-assisted process, and most recently using green chemistry technology (Song and Kim, 2009). The green synthesis approach provides most advantages over the chemical and physical method as it is cost effective, eco- friendly and easy to scaled up for large-scale synthesis without applying energy, high pressure, temperature and toxic chemicals (Albrecht et al., 2006).

There are several reports on synthesis of green silver nanoparticles (AgNPs) using various plant products. However, there is still a need for economically stable, commercially viable and environmentally clean route to synthesize AgNPs by using new plant sources (Chung et al., 2016). During recent years, the use of plants and plant based product in the synthesis of various metal nanoparticles has been broadly investigated (Arunachalam et al., 2013). A number of ethno medicinal plants are industrially used for production of different herbal medicine as well bioactive compounds for healthcare and nutritional product. Among them, *P. serratum* is widely used throughout the world for its active pharmaceutical compounds curing of different gastrointestinal disease and strong antimicrobial agents. Different parts of the *P. serratum* plant have been effectively utilized as fruit, food and as potential therapeutic agents in traditional medicines (Panda, 2014; Panda et al., 2016). A number of phytocompounds, such as polyphenols, flavonoids, tannin, sugars, alkaloids and triterpenoids/steroids have been reported to be present in the *P. serratum* plant and its various parts including leaves, roots, fruits, seeds and others., which are responsible for potential antioxidant, anti-inflammatory, larvicidal and other medicinal properties (Tanamatayarat, 2016). Thus we used the leaves of *P. serratum* for the synthesis of metal nanoparticles which would be a lucrative, cost-effective and an eco-friendly approach.

Now a day, food spoilage is very common problem throughout the world due to the notorious activity of food borne pathogens (Soylu et al., 2009; Newell et al., 2010; Tajkarimi et al., 2010; Negi, 2012). Moreover, the development of new resistant pathogenic strains of bacteria to current available antibiotics has become a serious threat to the public health, which triggers the immediate development of strong new generation bactericides (Rai et al., 2009). As the food is very indispensable materials to the living beings, it is urgent necessary to think about the food safety, quality and increase the shelf life of it by unraveling new antimicrobials and antioxidant agents. There are many positive influences of AgNPs to be utilized as efficient antimicrobial agents. They are highly competent against a broad range of pathogenic microbes and parasites, with low systemic toxicity toward human (AshaRani et al., 2009; Abbasi et al., 2016). Besides, AgNPs have been proclaimed to be employed and tested for numerous biomedical and industrial applications including avoidance of bacterial colonization and eradication of microbes on different metal and non-metal medical devices, disinfectant agent in wastewater treatment plants, and in silicone rubber gaskets for preserving and transporting food and textile fabrics materials (Patra and Baek, 2017). As silver has long been known to exhibit a strong toxicity to a wide range of microorganisms, it is a great advantage to utilize silver based compound for antimicrobial applications against food borne pathogens as well as antioxidants to maintain the food quality.

The present study reported the biological synthesis of AgNPs using the cell-free aqueous leaf extract of *P. serratum* and evaluation of its potential application as an antibacterial agent against two Gram negative (*Pseudomonas aeruginosa* MTCC 2453 and *Escherichia coli* MTCC 739) and two Gram positive (*Bacillus subtilis* MTCC 736, *Staphylococcus aureus* MTCC 2940) food borne pathogenic bacteria along with antioxidant potentials in terms of DPPH and OH radical scavenging activity. Moreover, the cytotoxicity test against L-929 cell line (normal fibroblast) was carried out to evaluate the biocompatibility as well as multifunctionality for potential pharmaceutical and biomedical applications. Usage of these plant materials in the green synthesis of metal nanoparticles could proficiently prove the cost effective approach.

## Materials and Methods

### Collection and Preparation of Plant Extract

Healthy leaves of *P. serratum* were collected from forest of Similipal Biosphere Reserve (21°–28′ and 22°–08′ North latitude and 86°–04′ and 86°–37′ East longitude), Mayurbhanj, Odisha, India during the months of January to March 2015. The identified plant specimen was deposited in the Department of Botany, North Orissa University. The shed dried leaves were powdered and sieved using a 20-mm mesh in order to maintain a uniform size. To make aqueous leaf extract, 5 g of leaf powder was mixed with 50 ml of sterile distilled water and sonicated for 15–20 min. The sonicated aqueous extract was purified by repeated centrifugation. The purified extract was filtered through Whatman filter paper no. 40 and the filtrate was stored at 4°C for further use.

### Biosynthesis of Silver Nanoparticles (AgNPs)

For the biosynthesis of silver nanoparticles, the suitable reaction mixture was prepared by adding 1 ml of aqueous leaf extract and 9 ml of 1 mM AgNO_3_ solution in a clean 25 ml Erlenmeyer flask. On the contrary, same experimental set up of 1 ml of aqueous leaf extracts with 9 ml distilled water was kept as control. Both flasks were incubated for 2–4 h in the rotary shaker under dark conditions at 25°C. Later, the synthesized silver nanoparticles (AgNPs) were separated and purified by continuous centrifugation (9000 rpm; 20 min; 10°C) with sterile miliQ water. The dried AgNPs were kept at 4°C for further characterization and bioactivity study (Mohanta et al., 2016a).

### Characterization of Silver Nanoparticles

The biosynthesis of the silver (Ag) nanoparticles (bio reduction of the Ag^+^ ions) in aqueous solution was monitored periodically in UV-Vis spectrophotometer (Lambda 35^®^ PerkinElmer, USA) within the range of 400–600 nm. The UV–visible spectra of the resulting reaction solution was monitored as a function of reaction time at a resolution of 1 nm room temperature (25°C). The average size and surface charge of the silver (Ag) nanoparticles were analyzed by Zetasizer (ZS 90, Malvern, UK). The purified samples were 10-folds diluted with the phosphate buffer saline PBS (0.15M, pH 7.2). The aliquots were later sampled in dynamic light scattering (DLS) cuvettes and examined for equivalent diameters, size distribution and zeta potential. The particle diameters were assessed at scattering angle of 90° at room temperature (25°C). Fourier Transform Infra-Red spectra of the silver (Ag) nanoparticles were studied in FT-IR spectrophotometer (8400S, Shimadzu, Japan) in transmission (%) mode with a 200 scans. The AgNPs were pelletized with potassium bromide (KBr) having 1% sample concentration (w/w) and was analyzed against the background of pure KBr pellet.

The nano-scale size of silver particles were confirmed by analysis of morphological structure under scanning electron microscope (Jeol 6480LV JSM, USA) performed at acceleration voltage of 15 KV (Mohanta and Behera, 2014; Nayak et al., 2015).

### Antibacterial Activity against Food Borne Pathogens

#### Microbial Strains

Common food borne pathogens viz. *B. subtilis* (MTCC 736), *S. aureus* (MTCC 2940), *P. aeruginosa* (MTCC 2453), and *E. coli* (MTCC 739) were used for the tests of antibacterial assay. All strains were procured from Microbial Type Culture Collection, Chandigarh, India.

#### Agar Well Diffusion and Micro Broth Dilution Methods

A single colony of each bacterial strain was inoculated from an agar slant in 1 mL Muller Hinton broth medium (0.2% beef extract, 0.015% soluble starch and 1.75% casamino acids) under aseptic conditions. The reaction tubes were incubated overnight (200 rpm; 37°C).

The antibacterial activities of AgNPs were investigated against bacterial species using well diffusion method on Muller Hinton Agar. To test the antibacterial activity, Muller Hinton Broth culture (100 μl) of each test organisms were seeded over the Muller Hinton Agar plates. Wells were made of approximately 5 mm in diameter and 2.5 mm deep. Each well was filled with 50 μl of AgNPs. Simultaneously, 50 μl of AgNO_3_ solution was kept to serve as control while standard antibiotic Gentamicin was used as a reference. The plates were incubated at 37°C for 24 h. After the incubation period, the diameter of the growth inhibition zones was measured. The AgNPs with the zone of inhibition greater or equal to 8-mm diameter were regarded as the positive activity.

Further, the confirmatory antibacterial activity was observed through micro broth dilution method along with calculation of the minimum inhibitory concentration (MIC) of AgNPs on bacterial strains (Panda et al., 2016). The percentage of inhibition more than 90% in micro broth dilution method was considered as potential activity and further experiments were conducted to calculate the MIC. Briefly, for MIC calculation, the test inoculum (190 μL; *A*_600_ = 0.1) with different concentrations of AgNPs (10 μL) ranges from 500 to 31.25 μg/ml (twofold dilution) were taken until the percentage of inhibition was found to be <50%. The micro broth dilution study was conducted in 96-well plates and the microbial growth or inhibition was measured in Microplate Reader (Biorad, USA) at 600 nm. The MIC was calculated by IC_50_/IC_90_ Laboratory Excel Calculation Tools and expressed as IC_50_. All the experiments were conducted in triplicates and the zone and percentage of inhibitions were expressed in mean ± SD.

### Qualitative Phytochemical Analysis

The qualitative phytochemical analysis of *P. serratum* extract was performed following the standard method (Parekh and Chanda, 2008; Arunachalam et al., 2012). The obtained results were qualitatively expressed as positive (+) or negative (-) Guruvaiah et al., 2012). The chemicals and reagents used for the study were purchased from Sigma–Aldrich (India).

### Quantitative Phytochemical Analysis and *In vitro* Antioxidant Properties

#### Total Phenolic Content Determination

Total phenolic quantity in the leaf extract was measured using Folin–Ciocalteu method with slight modifications (McDonald et al., 2001). All the experiments were performed in triplicates. The TPC was expressed as gallic acid equivalent (GAE) in mg/g sample.

#### Total Flavonoids Content Determination

Total amount of flavonoids were estimated by a modified aluminum chloride method (Chang et al., 2002). All estimations were carried out in triplicate. The TFC was expressed as GAE in mg/g sample.

#### DPPH Radical Scavenging Activity

Potential antioxidant activity was determined using 1, 1-diphenyl-2-picryl-hydrazil (DPPH) assay with sufficient modification wherever it seemed necessary (McDonald et al., 2001). Various concentrations, such as 5, 10, 15, and 20 μg/ml of AgNPs were taken for study of DPPH scavenging capacity. The MIC was calculated and results were presented IC50 value. The results were expressed as percentage (%) radical scavenging activity. The equivalent concentrations of ascorbic acid were taken as a positive control.

#### Hydroxyl Radical Scavenging Activity

The method was adapted with slight modification as reported by Tanamatayarat (2016). Fifty percent of the inhibitory concentration (IC_50_) was calculated from the percentage of scavenging capacity. Ascorbic acid was taken as a positive control. Different concentrations such as 20, 40, 60, 80, 100, 120, and 140 μg/ml of AgNPs and Ascorbic acid were taken for OH scavenging capacity and MIC determination.

### Biocompatibility Study

The biocompatibility of AgNPs was evaluated by calculating % of viability of cells by treating AgNPs on L-929 normal fibroblast cell line. The L-929 cells were seeded in flask with Dulbecco’s Modified Eagle’s Medium (DMEM) and M-199 medium supplemented with 10% fetal bovine serum (FBS) and incubated at 37°C (5% CO_2_) for 24 h. Following the incubation period, the attached cells were trypsinized for 3–5 min to get the individual cells and centrifuged (800 rpm, 10 min.). The cells were counted and distributed in 96 well Enzyme-linked immunosorbent assays (ELISA) plate with 5000 cells in each well and incubated for 24 h to form ∼70 to 80% confluence as a monolayer (Nayak et al., 2015). The AgNPs have the capacity to strongly reduce the Adenosine Triphosphate (ATP) content of the cell which ultimately cause mitochondrial damage and increase the production of reactive oxygen species (ROS) in a dose-dependent manner (Nayak et al., 2016). Hence the toxicity of AgNPs was determined at different concentrations ranges from 100 to 700 μg/ml in triplicates. To detect the cell viability, 3-(4, 5-dimethylthiazol-2-yl)-2, 5-diphenyltetrazolium bromide (MTT) solution 200 μl was added to each well and left for incubation (4–5 h). Later, the MTT solution was discarded and 200 μl of DMSO solvent was added to each well under dark followed by 15–20 min. of incubation and later the optical density (OD) of the formazan product was measured at 595 nm in a micro-triter plate reader (Biorad, USA) (AshaRani et al., 2009). The media, antibiotics and other chemicals used in these experiments were purchased from Sigma–Aldrich (India).

### Statistical Analysis

Each activity assay was performed in triplicates in order to determine their reproducibility. The antioxidant results were expressed as percentage of inhibition whereas the cytotoxicity results were represented as percentage of viability with respect to control values. The values of antioxidant and cytotoxicity assays results were compared by Student’s *t*-test with their control values. The antibacterial data were subjected to analysis of one way ANOVA and Duncan’s Multiple Range Test using the SPSS statistics program (IBM SPSS statistics 19). A significant difference was considered statistically significant at *p* ≤ 0.05.

## Results And Discussion

### Biosynthesis and UV-vis Spectra Analysis of AgNPs

**FIGURE 1:**
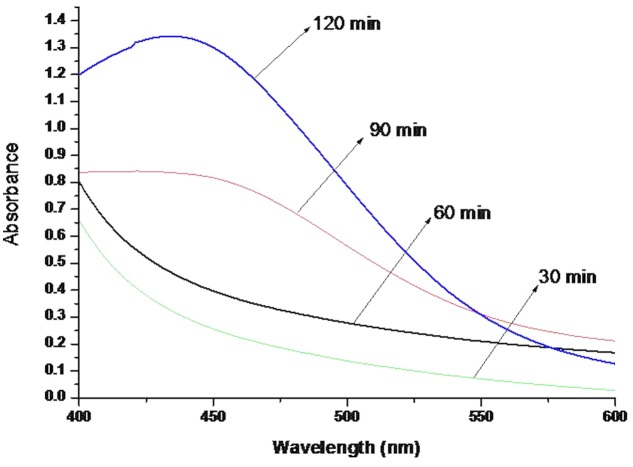
**UV-Vis spectra of AgNPs synthesized by *P. serratum***.

The UV–vis spectroscopy is an indirect method to examine the bioreduction of Ag nanoparticles from aqueous AgNO_3_ solution. Initially 9 ml of 1mM AgNO_3_ solution was taken for the bioreduction of silver by aqueous leaf extract. Two hours post-addition of leaf extract to the AgNO_3_ solution, a visible color change was observed from pale yellow to dark brown. The intensity of the color increased with increase in incubation time due to the excitation of surface plasmon vibrations in the metal nanoparticles (Jain et al., 2007). The AgNPs synthesized by *P. serratum* extract exhibited characteristic peak at 432 nm. Previous studies reported that the silver ions give absorption in between 430 and 440 nm due to its surface plasmon resonance (Chung et al., 2016). The AgNPs from *P. serratum* extract has shown peak at 432 nm which confirms the biosynthesis Ag nanoparticles (**Figure 1**). In the present study, the Ag nanoparticles was observed to be very stable in the solution, even after 6 months of their synthesis, which strongly validates the use of aqueous leaf extract of *P. serratum* in synthesis of AgNPs. The *P. serratum* leaf is rich in flavonoids, sugar, phenolic compounds, tannins and terpenoids, which contribute to its distinct aroma (Tanamatayarat, 2016). The terpenoids were believed to play an important role in biosynthesis of AgNPs through the reduction of Ag ions to its elemental form. Shankar et al. (2003) reported about the possible role of terpenoids from *Geranium* leaf in the synthesis of nano-sized Ag particles (Shankar et al., 2003). Polyols such as terpenoids, flavones and polysaccharides in the *Cinnamomum camphora* leaf were reported to be the main cause of the bioreduction of silver and chloroaurate ions (Huang et al., 2007). A similar mechanism might have operated in the present case as well where the flavonoids and phenolic compounds extracted from *P. serratum* leaf might have act as capping and stabilizing agents. To summarize these results, the water-soluble fractions comprised of complex polyols (Sharma et al., 2009) in the biomass were believed to have played a major role in the bioreduction of Ag ions.

### DLS Analysis

**FIGURE 2:**
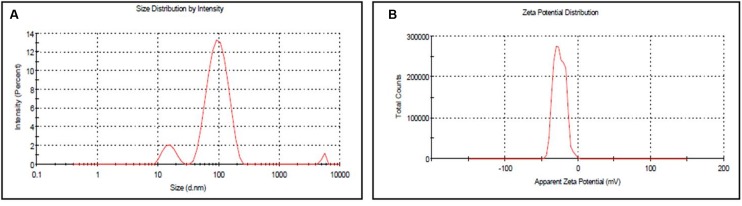
**(A)** Size distribution of synthesized AgNPs **(B)** Zeta potential of synthesized AgNPs by DLS analysis.

The size distribution and surface charge of the AgNPs were determined using DLS in aqueous solution. It was found that the average size and charge of the AgNPs were 74.56 ± 0.46 nm and −25.0 ± 7.84 mV, respectively (**Figures 2A,B**). The average size and potential contribute a strong characteristic of AgNPs to be used in biomedical sciences. The size of the particle is very important in cellular transportation. Smaller the size, it is easier to pass through the plasma membrane of the cell. So the nano size particle <100 nm was considered to be useful particles for different applications in drug delivery as well as in development of biosensors (Mukherjee et al., 2014). Besides the size of the AgNPs, the surface charge of the nanoparticles was considered to be important for interaction with different macromolecules as well as biochemical pathways present in the cell (Nayak et al., 2015).

### FTIR Spectroscopic Analysis

**FIGURE 3:**
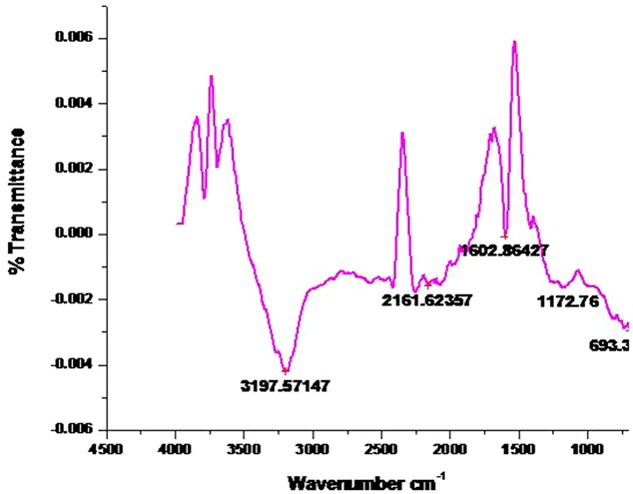
**Fourier transform infrared spectroscopy result analysis of AgNPs synthesized by *P. serratum leaf*.** *X*-axis represents the spectra (cm^-1^) and *Y*-axis represents percent (%) transmission.

The FTIR spectra of the AgNPs was recorded in order to identify the functional groups of the biomolecules present in the aqueous extract of *P. serratum* leaf involved in the synthesis and stabilization of the nanoparticles. The interaction of nanoparticles with phytochemicals of *P. serratum* showed intense peaks at 3197.57; 2161.62; 1602.86; 1172.76 and 693.3 cm^-1^ (**Figure 3**). A strong absorption peak was found at 3197.57 cm^-1^ strongly suggested the binding of silver ion with hydroxyl group and the broad spectrum at 2161.62 cm^-1^ was referred as the strong stretching of -OH group. The other three bands ∼1602.86 cm^-1^, ∼1172.76, and ∼693.3 cm^-1^ were due to stretching vibrations of *C* = O, C-C, C-N and O-H functional group, respectively. The *C* = O and C-N stretching are generally found in the proteins involve in the reduction of the metal ions. The observations suggested that the hydroxyl and carbonyl groups might be responsible for the synthesis of AgNPs.

Overall, it can be concluded that the *P. serratum* proteins adsorb as a layer over the green-synthesized silver nanoparticles, which stabilized them. It is well known that the proteins can bind to the silver nanoparticles via free amine groups or through cysteine residues in the saponins, phenolic and quinones from *P. serratum*, thereby stabilizing the nanoparticles formed through the surface-bound proteins. The present result is strongly supported by previous reports as well (Pant et al., 2012; Saranyaadevi et al., 2014).

### SEM Study

**FIGURE 4:**
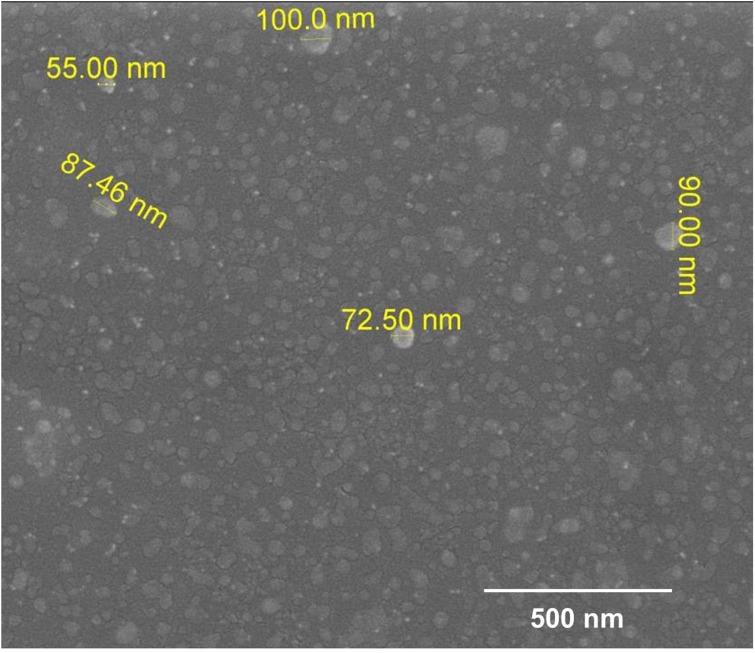
**Scanning electron microscopy image of biosynthesized AgNPs**.

The morphology along with spherical shape and monodispersity nature of the synthesized nanoparticles capped with its biomoities were confirmed by the SEM micrograph (**Figure 4**). Almost all nanoparticles were irregularly spherical with smooth edge. The shape of the nanoparticles had profound impact during the conjugation with specific drug molecules and target to the cells (Dauthal and Mukhopadhyay, 2016).

### Antibacterial Activity of Silver Nanoparticles

**FIGURE 5:**
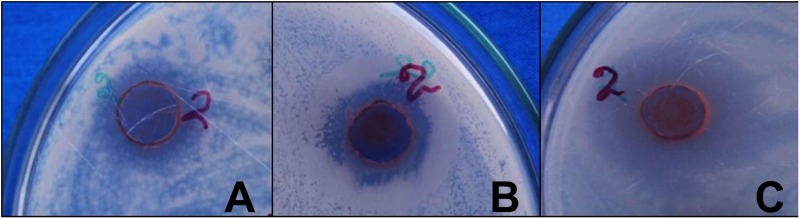
**Antibacterial activity of AgNPs synthesized by *P. serratum* against (A)** *B. subtilis* **(B)** *E. coli* **(C)** *P. aeruginosa* (where 2 = PS-AgNPs).

Preliminary screening of antibacterial activity was evaluated by agar well diffusion method against four pathogenic bacteria reported in **Table 1**. In agar cup method, zone of inhibition was found against Gram positive bacteria *B. subtilis* and Gram-negative bacteria and *E. coli*, and *P. aeruginosa* (**Figure 5**) while no zone of inhibition was found against *S. aureus*.

**Table 1:** Antimicrobial activity of AgNPs by agar-well diffusion method.

	Mean zone of inhibition ± SD (in mm)		
	
*Name of the test strain*	*Silver nanoparticles (500 μg/mL)*	*Gentamicin (10 μg/mL)*	*Silver nitrate (500 μg/mL)*
*Bacillus subtilis*	13 ± 0.50	20.8 ± 0.59	0
*Staphylococcus aureus*	0	16.3 ± 0.22	0
*Escherichia coli*	13 ± 1.00	13.13 ± 0.13	0
*Pseudomonas aeruginosa*	22 ± 1.15	15.3 ± 0.22	0

The micro broth dilution assay was followed to verify the antibacterial activity of AgNPs and the percentage (%) of inhibition and MIC of each strain is reported in **Table 2** (Supplementary Data Sheet 1). All the three strains showed growth inhibition above 99%. The MIC was calculated in terms of IC_50_ value and found to be effective against *P. aeruginosa* (74.26 ± 0.14 μg/ml), *E. coli* (84.28 ± 0.36 μg/ml) and *B. subtilis* (94.43 ± 0.42 μg/ml) (**Table 2**). The specific mechanism of nanoparticle-mediated antibacterial activity is not clearly understood till date. However, various probable mechanisms were explained in the literature regarding the antibacterial effect of nanoparticles. Sondi and Salopek-Sondi (2004) proposed that nanoparticles penetrate the cell wall of the bacteria due to their anchoring ability which ultimately responsible for the structural changes of the membrane and finally force to cell death (Sondi and Salopek-Sondi, 2004). There are several probable prospective mechanisms are exists for the decisive antibacterial activity of AgNPs which comprise the enzyme degradation, inactivation of major cellular proteins and impairment of genetic materials (Guzman et al., 2009, 2012; Patra and Baek, 2017). Numerous bacterial enzymes are inactivated due to firm interaction of Ag ions, released from silver nanoparticles with –SH groups which is the major structural part of the enzyme conformation (Yousefzadi et al., 2014; Swamy et al., 2015). Frequent interaction of AgNPs with the sulfur and phosphorus groups, intrude in the DNA replication and subsequently disintegrate microbial system (Singh et al., 2014; Ramesh et al., 2015). However, continuous in depth research is highly needed to prove the exact mechanisms about the antibacterial activity of nanoparticles. Such antibacterial properties signify AgNPs as possible candidate for the pharmaceutical industries in advancement of contemporary antimicrobial products. Moreover, the AgNPs could be convenient for formulating the polymeric materials for food packaging and other useful durable antimicrobial proof materials.

**Table 2:** Antimicrobial activity of AgNPs by micro broth dilution method.

					Antibacterial activity of AgNPs [Percentage of inhibition (%) ± SD]

**Name of the test strain**	**500 μg/ml**	**250 μg/ml**	**125 μg/ml**	**62.5 μg/ml**	**31.2 μg/ml**	**IC_50_ (μg/ml)**
*Bacillus subtilis*	99.6 ± 0.16^a^	99.4 ± 0.04^a^	60.5 ± 0.70^b^	34.5 ± 0.61^c^	1.67 ± 0.47^d^	94.43 ± 0.42
*Escherichia coli*	99.3 ± 0.08^a^	99.4 ± 0.08^a^	69.87 ± 0.33^b^	34.9 ± 0.04^c^	3.0 ± 0.81^d^	84.28 ± 0.36
*Pseudomonas aeruginosa*	99.3 ± 0.08^a^	99.3 ± 0.16^a^	65.1 ± 0.08^b^	45.0 ± 0.08^c^	5.6 ± 2.1^d^	74.26 ± 0.14

*The data are expressed as a percentage inhibition of bacteria and represent the mean ± SD (*n* = 3). Antimicrobial activity: exponentially growing cells were treated with different concentrations of AgNPs for 24 h and cell growth inhibition was analyzed through broth dilution assay. In each row, mean values followed with different superscripts significantly differ from each other according to Duncan’s Multiple Range Test (*P* < 0.05). IC_50_ is defined as the concentration, which results in a 50% reduction in cell numbers as compared with that of the control cultures (AgNPs). The values represent the mean ± SD of three individual observations.*

### Qualitative and Quantitative Assessment of Phytochemicals and Corresponding Anti-oxidative Activities

Qualitative and quantitative phytochemical examinations of the aqueous leaf extracts has summarized in **Tables 3**, **4**. The phytochemical analysis revealed the existence of flavonoids, tannins, phenolic, sugars and triterpenoids whereas glycoside, steroids and sterols were found to be absent. The phytochemical study of the leaf extract of *P. serratum* showed that flavonoids, tannins, phenolic compounds, sugars were present in the extract which may be the principal chemicals constituents responsible for the synthesis of AgNPs. Shankar et al. (2003) reported the possible of role of terpenoids from *Geranium* leaf in the synthesis of nano-sized Ag particles (Shankar et al., 2003). Polyols such as terpenoids, flavones and polysaccharides in the *C. camphora* leaf were reported to be the main cause of the bioreduction of silver and chloroaurate ions (Huang et al., 2007).

**Table 3:** Qualitative phytochemical screening of aqueous extract of *P. serratum*.

Name of the phytoconstituents	Observation
Alkaloids	-
Tannins and phenolic compounds	+++
Glycoside	-
Flavonoids	+++
Steroids and sterols	-
Triterpenoids	+
Sugars	+++

*“+++”, Highly present; “+”, Less present; ‘-’, Absent.*

**Table 4:** Quantitative phytochemical constituents of aqueous extract of *P. serratum*.

Phytochemical constituent	mg/100 g dry weight (Mean ± SD)
TPC	530.57 ± 26.00
TFC	810.76 ± 25.10

The current work does not report the presence of glycosides, steroids and sterols which might be an outcome of selective qualitative test performed, and/or extraction procedures. As long as the hypothetical mechanism of AgNPs’ biosynthesis is concerned, cascades of complex antioxidant enzymes might be involved in the biosynthesis of Ag nanoparticles (Prasad, 2014).

**FIGURE 6:**
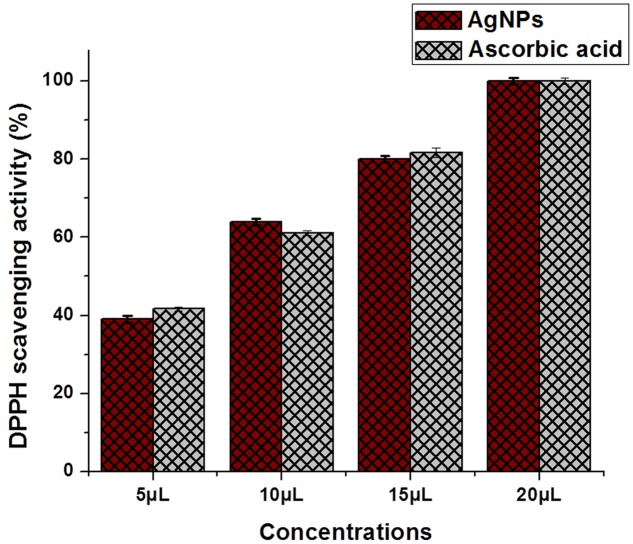
**1, 1-diphenyl-2-picryl-hydrazil radical scavenging assay of AgNPs.** The data are presented in the form of a bar graph and plotted using mean ± SD of individual replicates (*n* = 3). The *P*-value for significantly different mean, *P* > 0.05 versus positive control (ascorbic acid).

**FIGURE 7:**
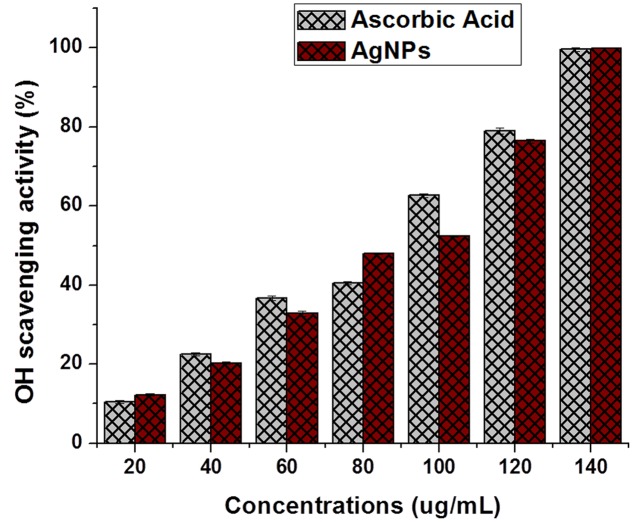
**Hydroxyl radical scavenging assay of AgNPs.** The data are presented in the form of a bar graph and plotted using mean ± SD of individual replicates (*n* = 3). The *P*-values for significantly different mean values, *P* > 0.05 versus positive control (ascorbic acid).

Antioxidant potential result for *P. serratum* exhibits a positive response toward the possible involvement of antioxidant molecules from the leaf extract during the biogenic synthesis of AgNPs. It is known that, the plants have a large collection of phenolics and flavonoids which might have possess super anti-oxidative capabilities and considered strong free radical scavengers. Significant anti-oxidant activity was also observed by DPPH and hydroxyl radical scavenging assays (**Figures 6**, **7**). The antioxidant capacity was found to be due to DPPH scavenging activity (IC_50_= 6.78 ± 0.15 μg/ml) and hydroxyl radical assay (IC_50_= 89.58 ± 1.15 μg/ml). The presence of moderate concentration of total phenolics and flavonoids in *P. serratum* leaves indicated a notable anti-oxidant activity. The high molecular weight and the proximity of many aromatic rings and hydroxyl groups are more important for the free radical scavenging activity of bioactive compounds (Hagerman et al., 1998). Recently, Pratap Chandran et al. (2013) reported *in vitro* anti-oxidant potential of methanolic and aqueous extracts of *A. solanacea* Roxb. leaf through DPPH radical scavenging assay that strongly supports our present result. Luximon-Ramma et al. (2002) investigated the entire phenolic, proanthocyanidin, flavonoids and the anti-oxidant activities of vegetative and reproductive parts (Luximon-Ramma et al., 2002). The anti-oxidant activities were highly correlated with total phenolic levels. However, the result showed that the anti-oxidant activities of reproductive parts surpassed the anti-oxidant activities of the vegetative organs, including the pods that have the highest total phenolic and flavonoid contents (Abdel-Aziz et al., 2014). Related findings were also reported in the present experiments.

It is also essential to evaluate the anti-oxidant potential as some of the plant molecules are still remain with Ag nanoparticles after purification as a capping agent, which should not be harmful to cells during cellular application of the nanoparticles. Thus, the anti-oxidant potential of *P. serratum* established the green synthesis process of silver nanoparticles to be highly safe for biological applications.

### Cytotoxic Activity/Biocompatibility Study

**FIGURE 8:**
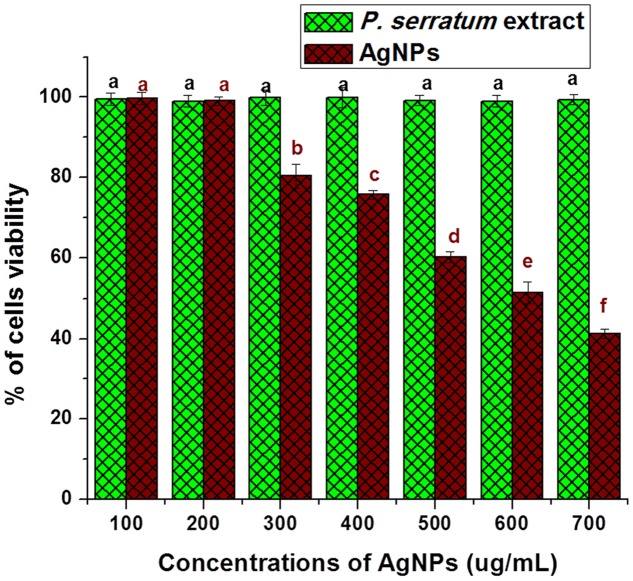
**Biocompatibility activity study of AgNPs on normal fibroblast cell line (L-929).** The data are expressed as a percentage viability of cells and represent the mean ± SD (*n* = 3). In all individual experiments, control (without treatment) was taken as 100% viable. Bars with different superscripts significantly differ from each other (*P* < 0.05). Bars with the same superscripts (*P* > 0.05) are not significantly differing from each other.

It is very pivotal to understand the biocompatibility of AgNPs for its successful implication in biomedicine and its direct use by human beings as food additives. The cytotoxicity of AgNPs was also tested against normal fibroblast cell lines L-929 to check their biocompatibility. The safety use of AgNPs is a major concern along with toxicity against normal cell lines which can impact on the biological applications. In the present study, AgNPs have not been observed of inhibition against L-929 cell line at lower concentrations. The percentage of cell viability of normal fibroblast cells is declined with an increase in concentration of AgNPs (**Figure 8**). The IC_50_ value of AgNPs against normal L-929cell lines was calculated as 600.28 ± 0.75 μg/mL. The IC_50_ value indicates the high biological compatibility and safe use of AgNPs in human body. The plant extract did not show any toxicity against L-929cell line and proved its potential use in synthesis of AgNPs. The AgNPs were also previously studied for its biocompatibility against Chinese hamster ovary (CHO) cell line (Netala et al., 2016). The study of Netala et al. (2016) corroborated with our present findings. In fact, the AgNPs should be thoroughly studied for its safety and biocompatibility before its practical application as product.

## Conclusion

Silver nanoparticles exhibited enormous antibacterial potency against three food borne pathogens. Such positive results highly recommend that AgNPs can be used in food packaging materials and also as disinfectant and cleaning agents. Further, the antioxidant activity of AgNPs revealed the protection from oxidation due to external factors as well as radical activity. The AgNPs were also very much stable and biocompatible to the human cell lines. Ethno-medicinal report suggests that *P. serratum* extract is not harmful to the human body and oral administration of its leaf extract is highly effective against gastrointestinal disorders and also stomach ulcer (Panda et al., 2016). Hence the present research highlights the potential involvement of nanoscience in food industry.

## Author Contributions

YM carried out all the experiment and wrote the manuscript. SP edited the manuscript. AB revised the manuscript. TM English editing and revised the manuscript.

## Conflict of Interest Statement

The authors declare that the research was conducted in the absence of any commercial or financial relationships that could be construed as a potential conflict of interest.
